# Supplementary material for: A Critical View of the Application of the APEX Software (Aqueous Photochemistry of Environmentally-Occurring Xenobiotics) to Predict Photoreaction Kinetics in Surface Freshwaters
Source: Molecules. 2019 Dec 18;25(1):9. doi: 10.3390/molecules25010009 (PMC7017383; doi:10.3390/molecules25010009)
Supplement: Supplementary file 1 [file molecules-25-00009-s001.zip › 3.2.4_gcc-4.4.0.zip!/3.2.4_gcc-4.4.0/3.2.4_gcc-4.4.0/doc/octave/html/interpreter/Exception-and-Error-Handling-in-Oct_002dFiles.html]

Exception and Error Handling in Oct-Files - Untitled


Next: Documentation and Test of Oct-Files,
Previous: Input Parameter Checking in Oct-Files,
Up: Oct-Files


---

#### A.1.12 Exception and Error Handling in Oct-Files

Another important feature of Octave is its ability to react to the user
typing `Control-C` even during calculations. This ability is based on the
C++ exception handler, where memory allocated by the C++ new/delete
methods are automatically released when the exception is treated. When
writing an oct-file, to allow Octave to treat the user typing `Control-C`,
the `OCTAVE_QUIT` macro is supplied. For example

```
     for (octave_idx_type i = 0; i < a.nelem (); i++)
       {
         OCTAVE_QUIT;
         b.elem(i) = 2. * a.elem(i);
       }
```

The presence of the `OCTAVE_QUIT` macro in the inner loop allows Octave to
treat the user request with the `Control-C`. Without this macro, the user
must either wait for the function to return before the interrupt is
processed, or press `Control-C` three times to force Octave to exit.

The `OCTAVE_QUIT` macro does impose a very small speed penalty, and so for
loops that are known to be small it might not make sense to include
`OCTAVE_QUIT`.

When creating an oct-file that uses an external libraries, the function
might spend a significant portion of its time in the external
library. It is not generally possible to use the `OCTAVE_QUIT` macro in
this case. The alternative in this case is

```
     BEGIN_INTERRUPT_IMMEDIATELY_IN_FOREIGN_CODE;
     ...  some code that calls a "foreign" function ...
     END_INTERRUPT_IMMEDIATELY_IN_FOREIGN_CODE;
```

The disadvantage of this is that if the foreign code allocates any
memory internally, then this memory might be lost during an interrupt,
without being deallocated. Therefore, ideally Octave itself should
allocate any memory that is needed by the foreign code, with either the
fortran\_vec method or the `OCTAVE_LOCAL_BUFFER` macro.

The Octave unwind\_protect mechanism (The `unwind_protect` Statement)
can also be used in oct-files. In conjunction with the exception
handling of Octave, it is important to enforce that certain code is run
to allow variables, etc. to be restored even if an exception occurs. An
example of the use of this mechanism is

```
```
     #include <octave/oct.h>
     #include <octave/unwind-prot.h>
     
     void
     err_hand (const char *fmt, ...)
     {
       // Do nothing!!
     }
     
     DEFUN_DLD (unwinddemo, args, nargout, "Unwind Demo")
     {
       int nargin = args.length();
       octave_value retval;
       if (nargin < 2)
         print_usage ();
       else
         {
           NDArray a = args(0).array_value ();
           NDArray b = args(1).array_value ();
     
           if (! error_state)
             {
               unwind_protect::begin_frame ("Funwinddemo");
               unwind_protect_ptr 
     	    (current_liboctave_warning_handler);
               set_liboctave_warning_handler(err_hand);
               retval = octave_value (quotient (a, b));
               unwind_protect::run_frame ("Funwinddemo");
             }
         }
       return retval;
     }
```
```

As can be seen in the example

```
     unwinddemo (1, 0)
     => Inf
     1 / 0
     => warning: division by zero
        Inf
```

The division by zero (and in fact all warnings) is disabled in the
`unwinddemo` function.
